# Paleopathology and Nutritional Analysis of a South German Monastery Population

**DOI:** 10.1155/2015/486467

**Published:** 2015-08-06

**Authors:** Andreas G. Nerlich, Alfred Riepertinger, Ralph Gillich, Stephanie Panzer

**Affiliations:** ^1^Division of Paleopathology, Institute of Pathology, Academic Clinics München-Bogenhausen and München-Schwabing, 81925 Munich, Germany; ^2^Department of Radiology, Murnau Trauma Center, 82418 Murnau am Staffelsee, Germany; ^3^Biomecanics Laboratory, Paracelsus Medical University Salzburg at the Murnau Trauma Center, 82418 Murnau am Staffelsee, Germany

## Abstract

The monastery of Attel, Upper Bavaria, which was founded in AD 1030, harbours a series of crypt burials from the time period between AD 1700 and 1750. Due to a restoration of the church, 16 crypts had to be removed and were subjected to an extensive anthropological-paleopathological and isotope analysis. The 16 crypts contained 19 burials in open wooden coffins. All bodies were covered by an extensive layer of calcium carbonate. Despite this “treatment,” bone and teeth were excellently preserved (mean degree of conservation > 75%, completeness > 85%). The anthropological investigation revealed a mean age of 38.5 years and a body height of 1.71 m. Paleopathologically, a surprisingly high rate of trauma was seen (13 injuries in 7 different individuals, i.e., 36.8% of individuals affected), 2 cases presented signs of extensive arthritis urica (gout), and several monks were affected by arthrosis of shoulder and knee joints. Extensive dental attrition, numerous foci of dental caries, and dentogenic abscesses coincided with considerable dental calculus indicating poor oral hygienic conditions. Stable isotope analysis showed adequate mixed carnivore-herbivore nutrition, comparable to that of contemporaneous upper class individuals. This extensive combined analysis provides considerable insight into the nutrition and disease pattern of a middle-class monastery of early 18th century South Germany.

## 1. Introduction

Most paleopathologic studies analyze human remains from defined spatial and temporal settings and try to deduce certain aspects of living conditions and diseases within this population. Surprisingly little attention is paid to selected populations, such as monasteries [1].

Monks represent a particular part of any past or present population. This holds true for various time periods and regions, although historic (mainly literary) evidence suggests differences in tasks, structure, and living conditions between various monasteries. Therefore, the multidisciplinary analysis of the human remains of monastery populations is of major interest.

Recent advances have clearly shown that the successful investigation of ancient human remains requires the application of a multitude of analytical techniques, depending on the available tissue and its degree of conservation, but also on the applicable techniques [2].

The present study was therefore designed to fill part of this gap by an interdisciplinary analysis of a population of very well-preserved skeletons from a South German “middle-class” monastery of a distinct time period between 1700 and 1750 AD. Since some of the individuals could be identified, the available monastery archive [3] helped us to reconstruct even individual disease histories.

## 2. Material and Methods

**Figure 1:**
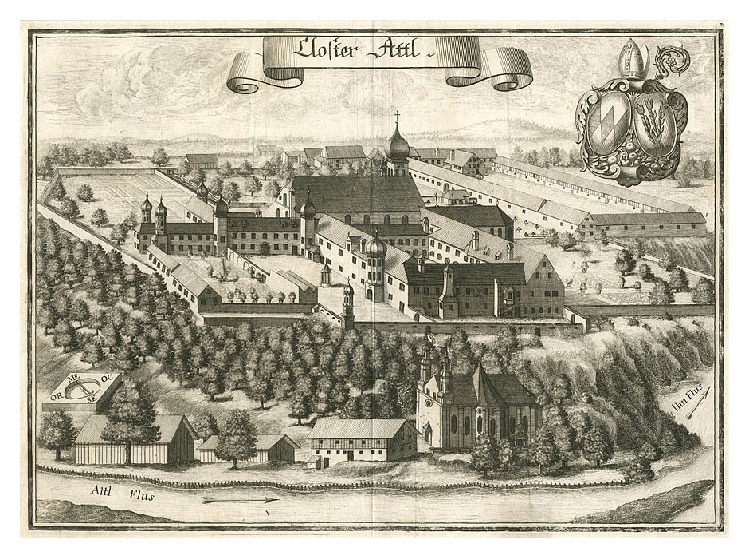
The monastery of Attel, Upper Bavaria, in 1700 (image: collection A. Nerlich).

In this study, we investigated the human remains from 19 individuals that have been recovered during a renovation project of the crypt of the monastery church of Attel. This small monastery which was founded in the year 1037 AD, destroyed in 1070 AD, and reopened in 1110 AD was continuously inhabited until the year 1803 AD (when all monasteries in Bavaria were closed due to secularization). It is located on the banks of the river Inn (Figure 1). Although the monastery was repeatedly damaged by flooding of the river Inn, this site was also advantageous for economic reasons, since the river provided a good opportunity as an efficient transport route for various goods. Accordingly, the written records document a moderate wealth of the monastery for several centuries. Besides the danger of flooding by the river Inn, recurrent warfare endangered the monastery for several times, such as what happened during the Thirty Years' War. However, in the period of “our” monastic population (i.e., between AD 1700 and 1750), there is no record of such an event. Accordingly, at this period, economy seems to have been stable and the support of monks and both employed and dependent people of the monastery should have been sufficiently good [3].

The records of the monastery, recently worked up into a large monograph [3], not only provide insight into the organization of daily life and the economic development but also give some detailed data on individual persons within the monastic community, including names, age at death, and function within the community, and also some hint on distinct diseases in certain individuals and eventually their cause of death. The burial place in the monastery church is below the church's altar and was reserved for monks. The crypt consists of 40 burial chambers that had been closed by brick walls. Although we assume that all chambers initially carried the names of the buried person, only a few of these inscriptions have been retained until today.

Since that church was completely renovated at the end of the 17th century, the burials cover a time period between approximately 1700 and 1750 AD, with later burial (i.e., after 1750 AD) having been performed on the newly formed cemetery close to the church. In this study, we only had access to 16 chambers which had to be removed for structural problems of the church, while the other 24 chambers remained unopened. On the wall of 4 of the chambers under investigation, an identification tag was present; 2 further individuals could be identified according to their precise location in one of the untagged chambers. All residual individuals remained unidentified.

After opening of the 16 burial chambers, all material was removed, prescreened into those residues of the (exclusively wooden) coffins, remains of clothes and grave goods, such as rosaries and crucifixes, cloths, and leather shoes, and the human remains. The open wooden coffins were mostly covered by large amounts of a whitish crumbly material that proved to be calcium carbonate and had obviously been used to “preserve” the cadavers and to avoid the undesired bad smell of the decomposing corpses following the burial. Despite this “treatment,” the skeletal elements (and teeth) proved to be well preserved. Soft tissue and hair, however, were present only very occasionally.

### 2.1. Evaluation of the Tissue Material

In the first step, all human biomaterial was cleaned, registered, and evaluated according to the presence of the skeletal elements (termed “representativity”) and the conditions of the bone substance (“preservation”). In order to standardize, we used the previously developed and applied scheme that covers 40 regions of the human skeleton and evaluates the presence of the respective skeletal material (0–40 points) and the condition of the bone (0–126 points) with low figures indicating an absent or very fragmented skeleton and an extremely poorly conserved bone substance, respectively [4]. In contrast, high figures indicate the presence of all/most skeletal elements and good/excellent preservation of the bone substance, respectively. This system has previously been successfully applied to other small skeletal series [5].

### 2.2. Anthropological Investigation

Following the above indicated evaluation, an anthropological survey of the bones provided data on sex, age at death, body height, and physiological characteristics of the individuals. Likewise, individual gender was determined on the pelvic suture and skull bone characteristics; age at death was calculated from skull bone sutures, symphyseal surface, and the ossification pattern of the first costosternal joints. Body height was estimated from the length of long bones according to established protocols. Physical properties, such as handedness, mobility pattern, and development of skeletal musculature, were determined from bone measurement ratios and extent of muscular insertion zones on long bones [6, 7].

### 2.3. Paleopathological Analysis

Features of pathological conditions were also determined as shown in previous studies, including sequels of trauma, chronic metabolic deficiencies, and dental pathology. Degenerative diseases of large joints and the vertebral column were evaluated using established criteria and standardized protocols [8].

### 2.4. Radiological Investigations

In order to enhance the paleopathological diagnostics, we performed X-rays and/or CT scans on isolated skeletal elements. Here, again, previous protocols were followed and the technical details of the analysis are given in [2].

### 2.5. Stable Isotope Analyses

For the investigation of the nutritional composition of the investigated individuals, we used standard bone samples from vertebral bodies of all identified persons. The analysis of nitrogen and carbon isotopes was performed according to established protocols [9]. Stable isotope analysis and concentration measurements of nitrogen and carbon were performed using ratio mass spectrometry (IsoAnalytical, Crewe, UK) as previously described in detail [2].

## 3. Results

### 3.1. Macroscopic and Anthropological Findings

**Figure 2:**
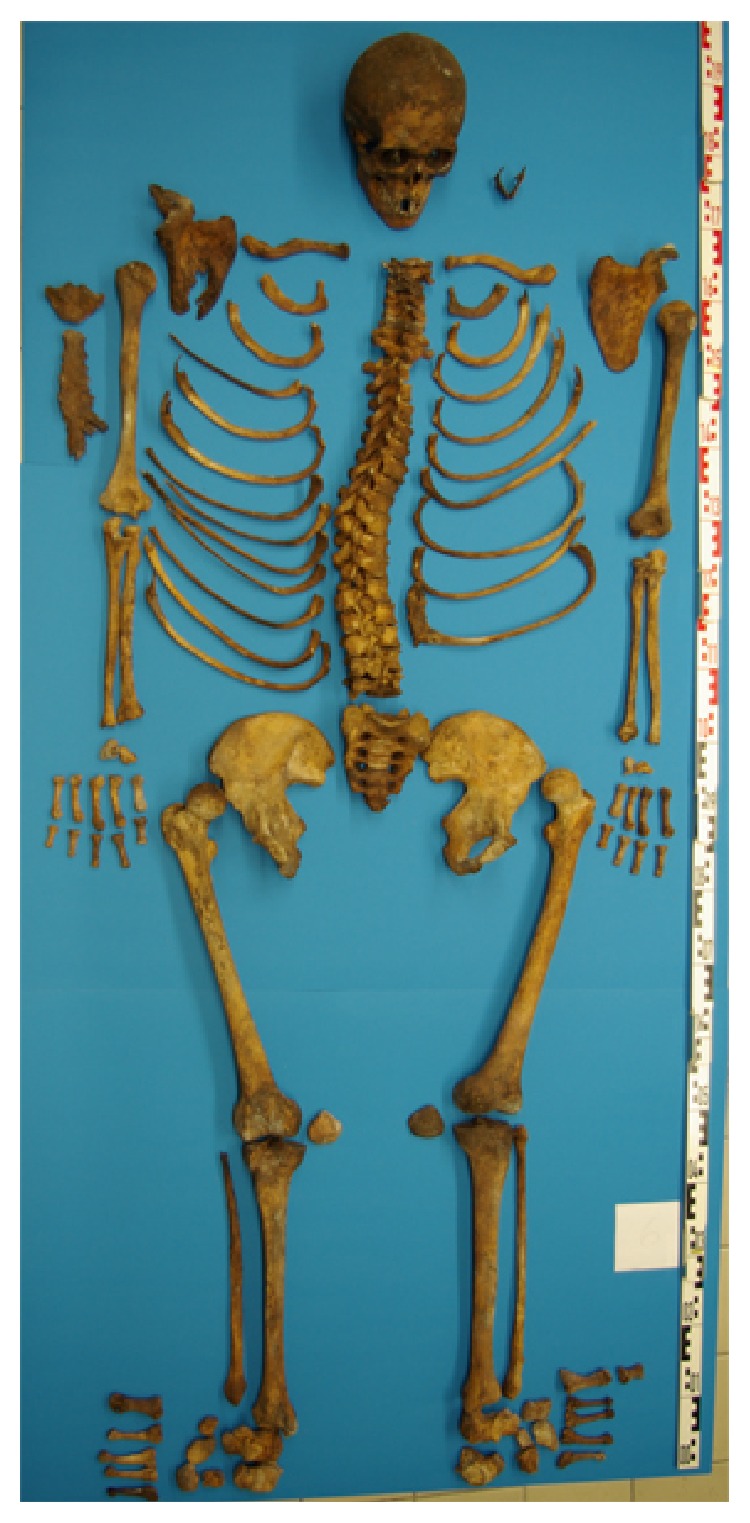
The skeleton of Gregorius Lechner (number 6) following removal from the burial chamber and cleaning. The skeleton is almost complete; the bone substance is excellently preserved.

In the 16 burial chambers, we were able to identify 19 individuals with double burials in 3 of the chambers. Generally, all bones were good to excellently preserved (Figure 2). The scoring of bone tissue preservation revealed values between 75 and 100% of maximum values (between 32 and 40 of 40 maximal points, average 81.9%). In parallel, the extent of skeletal representativity was also in most cases considerably high again reaching between c. 80 and 100% of maximum values (between 95 and 126 points of maximum 126 points, average 89.2%).

All individuals showed skeletal features of male persons. Most of them died in the 4th to 6th decade of age with 2 monks dying between 20 and 30 years, 7 individuals between 30 and 40 years, 6 persons between 40 and 50 years, and two cases reaching 50 to 60 and further two cases showing more than 60 years of age (on the average of 38.5 years). This age distribution correlates very well with the values that can be retrieved from written sources [3]. In a detailed evaluation, most monks died in the period between 1700 AD and 1750 AD at the age between 40 and 60 years and the resulting distribution curves are quite similar between the overall population of 37 Attel monks and the 19 individuals that we investigated. Furthermore, 6 persons were identified by either the inscriptions on the burial chamber walls and/or the written records. The anthropological age estimation correlates very well with the written data (*r* = 0.88).

Finally, certain physical properties could be identified. The body height ranged between 1.67 m and 1.78 m (on the average of 1.71 m) which is well above the values in various 18th to early 19th century populations in Southern Bavaria and neighbouring Austria where the average body height in males ranges at 1.62–1.64 m [10–12].

The determination of osteometric indices provides evidence for right handedness in 11 monks and left handedness in 7 persons. The mobility index (index platycnemius) reveals low mobility ratios (stenomeric). The muscular insertion zones were in all 19 individuals not significantly prominent and confirmed low physical activity profiles.

### 3.2. Paleopathologic Investigation

In 7 of the 19 individuals, the sequelae of trauma could be identified (36,8%). Some cases presented with multiple lesions which thereby summed up to 13 trauma lesions in those 7 affected individuals.

**Figure 3:**
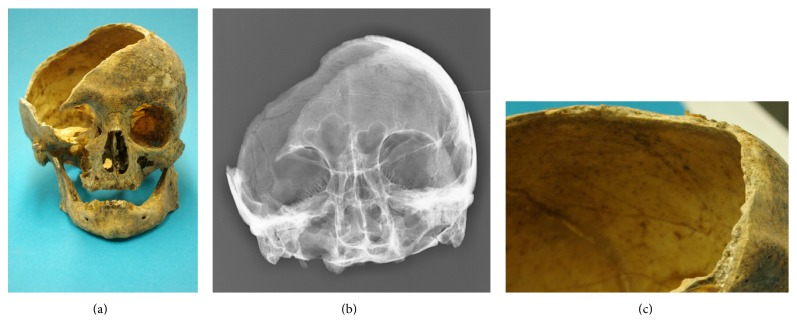
Massive defect of the skull bone suggesting severe trauma. The morphology indicates a massive attack by a sharp weapon, for example, a sword blow, without any bone reaction (a: macroscopy; b: radiology). A detailed aspect of the cut margin at the upper (temporal) skull bone (c) showing the brown colouring of the cut surface which runs from the outer table tangentially to the inner table such as in a sharp weapon blow.

These comprised one case with a large skull defect. This unique finding strongly suggests an attack with a sharp weapon such as a sword blow that had removed an almost 15 × 6 cm large piece of bone out of the temporoparietal region. This diagnosis is based on the lack of any adequate piece of bone in the skull region of the individual. In consequence, the piece of bone must have been removed from the skull before burial; that is, we see a premortem removal of that piece of bone. Furthermore, the dark brown colouring of the cutting margin indicates an “old” lesion, obviously occurring in previous time. Finally, the cut surface at the high parietal margin runs from the external bone table (tabula externa) to the internal layer of bone, while the margin at the lower temporal margins runs in the opposite way (Figure 3(c)), fitting best with a sharp weapon blow downwards. In summary, these findings strongly argue for an “old,” presumably perimortem, defect with typical cutting margins of a violent attack. This is also consistent with a perimortem time course, since the defect margins did not show active bone remodelling reaction (resorption and/or new bone formation) on X-rays and CT scans. This suggests a terminal and potentially lethal trauma (Figures 3(a) and 3(b)).

**Figure 4:**
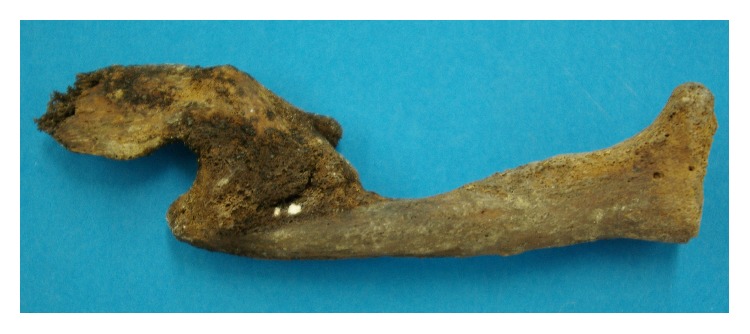
Old-healed fracture of the clavicle with fusion of the dislocated fracture ends.

In 3 cases, multiple old-healed fractures of several ribs were seen, in one case together with an also healed clavicle fracture suggesting massive trauma, for example, from falling from considerable height onto the thorax. In all cases, there were clear signs of new bone formation and callus-like bridging reaction of the defect zone. Further two cases revealed isolated healed clavicle fractures (Figure 4), one further case had an old-healed distal fracture of the radius, one case had a healed compression fracture of the right femoral condyle, and another case showed a torsion fracture of the ventral rim of the cervical vertebral body C7 without healing signs. Finally, 2 metacarpal bones had been fractured with extensive pseudoarthrosis and bone reaction and in two cases a complete unilateral fusion of the ileosacral joint suggests potential old-healed pelvic trauma.

In contrast to this high number of trauma residues, we had no case with cribra orbitalia (as a sign for chronic iron-deficiency and anemia) nor for chronic vitamin C-deficiency (scorbut). One individual presented with slight to moderate bowing of several long bones such as in mild infantile/juvenile rickets and one monk (of c. 30–40 years of age) showed a moderate loss of bone substance indicating osteopenia which is most presumably the result of prolonged vitamin D deficiency (osteomalacia) at that age.

**Figure 5:**
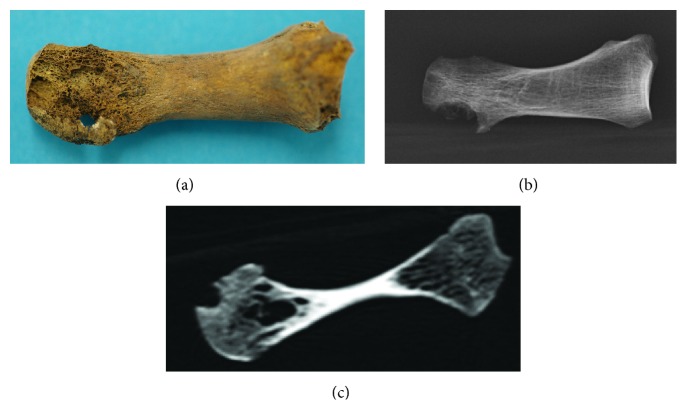
Severe osteolysis of the metatarsophalangeal joint in Gregorius Lechner indicating severe chronic gout (a: macroscopy; b: X-ray; c: CAT-scan).

Despite this very low number of metabolic osteopathies, 2 monks revealed the very typical signs of chronic hyperuricemia (gout) as evidenced from massive bilateral osteolytic destruction of the first metatarsophalangeal joint which was confirmed by typical intraosseous lytic lesions on X-rays and CT scans of the affected bones (Figure 5). In both cases, we were able to identify the respective historic person and were able to correlate the paleopathological findings with corresponding evidence from the written sources that had indicated chronic gout in both persons.

These two persons are here briefly described in more detail.

Gregorius Lechner (cf. Figures 2 and 5) was born on 25/02/1672 in a small Upper Bavarian village. He took his vows at the age of 23 and the ordination of priesthood at the age of 26 years. At the age of 41, he was designated the “Oeconomus major”; that is, he was responsible for all external economic transactions of the monastery. He was said to be of “weak healthiness” suffering from chronic gout and dropsy. At the age of 60, he died of both conditions. On paleopathological investigation, the most impressive finding was a severe bilateral arthritis urica of both first metatarsophalangeal joints, which was supplemented by a slight spondylosis of the lumbar spine, moderate osteoarthrosis of both shoulder joints, and severe arthrosis of both femoropatellar joints suggesting frequent and extensive kneeling position such as in frequent praying. Finally, significant oral pathology presented with extensive dental calculus formation and severe parodontosis, one apical dental abscess, and moderate dental abrasion.

The second affected individual, Bernhardus Veldhofer, was born on 17/11/1683 in the small town of Erding which is approximately 30 km from Attel monastery. He entered the monastic society at the age of 14 years and took his vows on 11/11/1706. Four years later he received the ordination as a priest. Having served first as a keeper of the monasteries gate, he rapidly proceeded to become the organizer of pilgrimages and was involved in the daily religious services for the surrounding rural population. The records describe him to be of weak health finally developing gout and dropsy which also were regarded as cause of death. He died on 24/5/1731 at the age of 48 years. The paleopathologic examination again revealed the very typical signs of gout with massive destruction of the first metatarsophalangeal joints such as seen in the skeleton of Gregorius Lechner. As the second most extensive pathology, the dental apparatus was affected with multiple teeth having been lost intravitally, two teeth with significant dental caries, and a large dentogenic abscess at the 26/27 region (upper left maxilla/teeth number 6 and 7) extending into the adjacent maxillary sinus and causing major remodelling of the osseous floor of this paranasal sinus. There was no evidence for major degenerative lesions of major joints or the vertebral column and also no signs of any other chronic disease affecting the skeleton nor any trauma sequelae.

The analysis of degeneration of large and small joints and that of the vertebral column revealed in several cases mild to moderate osteoarthrosis in the right shoulder joint and in both knee joints (according to the evaluation scheme by Schultz [8] average shoulder joint right 1.42 points versus left 0.89 points and right knee joint 1.24 points and left knee joint 1.38 points; all other values below 1). In parallel, the vertebral bodies (including the small facette joints) showed degeneration values ranging from 1.08 to 1.29 in the lumbar spine and from 0.43 to 0.92 points in the cervical and thoracal spine. Additionally, two cases presented with osteochondrosis dissecans of the knee joint.

As already indicated before, dental pathology was extensively present with moderate to severe dental abrasion, multiple cases with dental caries, and apical dental abscesses and also numerous cases with extensive dental calculus formation. As expected, those cases with dental calculus had lower frequencies of dental caries/dental abscesses and vice versa. The loss of teeth during lifetime was also a frequent event with only 4/19 cases without intravital dental loss and 5/19 cases with loss between 1 and 4 teeth, 3 cases between 5 and 9 teeth, 4 cases between 10 and 15 teeth, and 2 cases with a loss of more than 15 teeth (one with complete loss of dentition intravitam).

### 3.3. Stable Isotope Analyses

**Figure 6:**
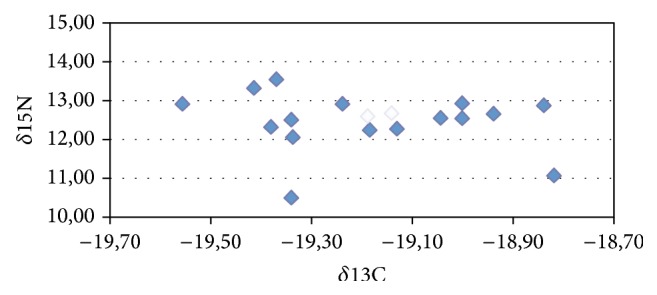
Isotopic results of the Attel monastery population. The two cases with paleopathological evidence for gout are indicated in open diamonds.

In order to further evaluate the nutritional status of this monastery population, we determined the ratios of stable nitrogen and carbon isotopes. As a result, there was a very similar nutritional pattern with nitrogen *δ*15N values ranging between 10.6 and 13.6 and carbon *δ*13C lying between −19.5 and −18.8. Most cases clustered closely together. Accordingly, the nutritional pattern is that of mixed carnivore-herbivore nutrition with considerable amounts of terrestrial protein and an adequate carbohydrate diet. There was no evidence for any dominant fish consumption. The two individuals with the massive gout were not different from their comonks (Figure 6).

## 4. Discussion

The circumstantial analysis of distinct past populations offers an excellent insight into ancient living conditions and diseases. To this regard, monastery populations represent a particular setting, as these reveal a selected group of individuals within historic populations.

In our present study, we investigated a series of 19 monks that had lived between AD 1700 and 1750. The anthropological estimation of the age at death very well correlates with the data from written sources providing evidence that our subpopulation may be quite representative for the complete monastic population that lived in the Attel monastery at that time. Furthermore, all individuals investigated proved to be males. These observations strongly support the notion that we investigated the genuine monastic population that has been well documented in written sources [3].

Most interestingly, the physical properties of the individuals showed a tall stature, particularly when compared to 18th-19th century reference populations of Southern Bavaria where the mean body size of males ranged at 1.62–1.65 m [10–12]. Accordingly, a detailed analysis of nonmonastic rural populations in Bavaria/Austria of the 18th/19th century shows lower values than in the Attel population [11, 12]. Even a contemporary Swedish population provides lower mean values (mean 1.67 m [13]) than the Attel monks, although it is well known that the body height in Northern (Scandinavian) individuals was larger than in Southern (Mediterranean) populations with Middle European populations ranging in between [14]. Since the written records indicate that most monks had entered the monastery society at juvenile age, this suggests a good nourishment and avoidance of major consumptive, for example, infectious, diseases during their monasticism. Accordingly, the mean body height in our population can be interpreted to reflect “better” living conditions [14]. However, life expectancy did not differ significantly from other rural populations in Bavaria, Northern Germany, and rural Poland [15–17] possibly due to the enhanced rate of metabolic diseases in the monastic population, for example, by the severe gout. Finally, our observation also very well correlates with the stable isotope values that indicate a balanced diet with sufficient protein supply mainly from animal source and good carbohydrate diet. Despite its close location to the river Inn, the fish consumption may not have dominated their daily food. Well in line with the sufficient food supply is the almost complete lack of metabolic malnourishment diseases, such as cribra orbitalia or lack of essential vitamins (see above).

The most interesting, but not surprising, finding is the unambiguous presence of 2 cases with severe chronic hyperuricemia (gout). Both had at least somewhat higher positions within the monastery society (Gregorius Lechner was even “oeconomus major” which means that he was the “chief” of all economic affairs of the monastery, Bernhardus Veldhofer obtained a less exposed position, but at least he was organizer of the pilgrimages and thereby he also must have had some influence on particular economic business). This suggests that they not only were well-maintained but also may have been subjected to excessive supply of alcohol, meat, and/or the consumption of protein from cell-rich internal organs, for example, liver.

Hyperuricemia is a chronic metabolic disease originating from a disturbance of the metabolism of purines characterized by the accumulation of its end product sodium urate within the body [1]. While a minor proportion of affected patients suffer from an inborn error of metabolism, in most instances hyperuricemia results from an excessive uptake of purines from the nourishment together with an enzymatic blockage of its degradation in the liver by toxic substances, such as alcohol and/or reduced excretion through the urinary system. The enhanced level of urate in the blood stream leads to its deposition in bradytrophic tissue, such as the capsule of peripheral small joints. This deposition occurs in crystal form thereby causing massive resorptive inflammation, with consequently swelling and redness of small joints, such as most preferentiallys the metatarsophalangeal and interphalangeal joints of the big toes. The chronic form of this condition leads to typical osseous resorption and joint destruction such as seen in our two present cases. The significant destruction of the first metatarsals further indicates that the metabolic condition in both affected individuals had persisted for considerable periods of time [1].

In paleopathology, chronic gout has repeatedly been identified dating back to ancient Egyptian mummies [18] where these findings could even be confirmed by laboratory investigations [19]. Furthermore, isolated cases have been seen in Roman cemetery findings [20] and mediaeval period skeletons [21], in several cases of the famous Medici family [22], but also as isolated findings or very small series in modern Pacific islanders [23]. Blondiaux et al. [24] described a familial accumulation of cases with arthritis urica in several skeletons of a French cemetery between the 7th century and the 18th century suggesting possible genetic links with living conditions in a historical privileged population. The most renown case of as yet paleopathologically verified gout is Emperor Charles V [25]. The investigation of one of his fingers provided circumstantial evidence for gout.

In general, however, there exist neither data on the prevalence of this disorder in antiquity, nor on its occurrence in specific populations such as monastic inhabitants although written sources suggest that gout was very frequent in history [26]. To this regard, our present analysis adds to the previous knowledge, since the observed severe gout in two persons with documented gout.

Besides these two cases with gout, we surprisingly found a high number of individuals with trauma sequelae with at least several cases typical for more or less massive trauma, such as falling from considerable height, but also minor trauma leading to broken metatarsals, and/or trauma from significant interpersonal conflict, such as a sharp weapon attack to the skull in one monk. These high figures highlight the dangerous daily life even within the monastic society, be it by accidental trauma or conflict situations. Unfortunately, no comparable data are available for any rural population in the region of that time period. However, in other 18th/19th century populations, trauma rates of rural population range up to 50% of individuals [26, 27].

Finally, massive pathology was seen in the dental apparatus with lack of oral hygiene and significant dental abrasion, most presumably by the consumption of food, for example, bread that had been prepared from cereals processed by stone mills. As a consequence, many monks suffered from caries, dental apical abscesses, and/or early intravital loss of teeth. In general, however, the high figures of dental pathology are not surprising in historic populations such as investigated here.

A comparison of the stable isotopic values with those of other historic populations from various regions and time periods indicates a similar level of nourishment between the Attel monks and Bavarian noblemen in terms of composition with adequate supply in terrestrial protein and carbohydrates. This is well in line with the written records that inform us that the Attel monastery of the early 18th century had a good economic basis. Furthermore, during this time we have indication that this economic stability must have not been endangered by warfare; the only major war at that time period was the “War of Spanish Succession” 1701–1714 which, however, did not affect the Attel region, nor by major environmental catastrophes, such as severe flooding by the river Inn.

In summary, our interdisciplinary study provides a further and extended insight into the living conditions of a very specific early 18th century monastic population, a new and profound example for the power of combined multidisciplinary historic studies that bring historic sciences together with natural historic ones.
